# Chronic Tobacco-Neurosis

**Published:** 1872-12-01

**Authors:** 


### CHRONIC TOBACCO-NEUROSIS.

TRANSLATED FROM THE GERMAN OF DR. ERLEMEYER, BY E. J. DOERING, HOUSE PHYSICIAN IN MERCY HOSPITAL, CHICAGO.

Although the acute symptoms of poisoning by tobacco and nicotina have been fully investigated, in the last few years, by Van den Broeck, Vleminks, Van Hassett, and others, still there are many dark points connected with the symptoms of chronic poisoning by tobacco, especially in regard to the nervous system. Some light on this subject will therefore be, perhaps, favorably received.

The author of these remarks has had occasion to observe several cases of chronic poisoning by tobacco, the most interesting of which he is going to relate.

R. S., a physician in a large city, about 44 years of age, and attending to a large practice, descends from a family in which diseases of the nervous system are of not unfrequent occurrence. He is a passionate smoker, and smokes particularly very strong cigars. Five years ago, up to which time he had been in perfect health, he was taken sick with blepharospasmus. This was increased by any physical exertion, even in speaking and chewing. Straining of the eye had the same effect. When he kept perfectly quiet, he was but little troubled. This spasm disappeared gradually, the patient not being able to state any reason of his improvement. In the summer of the following year, after having been very busy in the performance of his professional duties, he was suddenly attacked with vertigo, which lasted from five to six weeks, and forced the patient to remain in bed for ten days. The use of liquor, and entire rest for the body and mind, acted favorably; but every physical exertion increased the dizziness. During the night, he was but little troubled, and slept well.

This went on until the year following, when, in addition to the above symptom, the patient was attacked with muscular weakness, he being unable to stand any length of time. This gradually passed away.

A year later it again returned; and about the end of March pains in the head suddenly made their appearance, together with a feeling of anxiety and dizziness. The patient returned to his old remedies, and took a great amount of liquor to keep off the dizziness; but he gradually grew worse, and finally was obliged to give up his practice entirely. The following symptoms appeared about this time: he was very restless at night; he either slept only part of the night, or not at all, having often severe vertigo and great mental anxiety. Besides being troubled with photophobia which forced him to remain constantly in a dark room, he suffered with optical illusions. He was very sensitive to the least noise. Moreover, he was subject to extreme sensitiveness and timidity. Touching his bed would cause him to have headache. At times, the softest pillow would appear to him as hard as a rock. He was troubled, also, with different forms of neuralgia—as neuralgia supra-orbitalis, infra-orbitalis, temporalis, maxillaris, superior and inferior. After having been sick for five weeks, he was able to leave the city, and to go to a watering-place in South Germany. His residence here proved beneficial. In the beginning, he was able to walk with more security, slept better at night, and was less troubled with dizziness. During this period he had several small abscesses, following each other, on the right side of his face. After having remained here for fourteen days, in which time he did not take any medicine, but took a good deal of wine, and quit smoking, his condition was greatly improved. He was able to walk around all morning, without getting an attack of dizziness; the latter complaint only troubling him when he undertook to speak or read.

Some time after, he had to discontinue his morning walks, on account of the return of dizziness, which he ascribed to having taken a few cold baths.

It was at this stage of his disease that I saw him for the first time. In connection with the above symptoms, I ascertained two more facts. I found that whenever my patient walked on a smooth surface, he had the sensation of being on a floating vessel; and, furthermore, that he suffered with an enormous loss of sensibility, being equal on both sides of his body.

The views of the many colleagues whom our patient consulted in regard to the nature of his complaint, differed greatly. Some called it a disease of the brain, others of the spinal cord, and a few pronounced it to be a case of chronic poisoning by tobacco.

To state sufficient reasons for this latter view, is very difficult, as our pathological experience, up to this date, has not given us a thorough knowledge of (entire security in) the symptomatology of this disease. The views with regard to the injurious influence dependent on the manufacture of tobacco leaves in the fabrics, and their use in smoking, chewing, and snuffing, are greatly different. According to Foureroy, Painte, and others, the workmen in these fabrics are troubled with ophthalmia, skin diseases, headache, dizziness, colic, diarrhœa, asthma, and emaciation. Melier states that these workmen are all exposed to a certain degree of poisoning, especially those who are engaged in the drying and fermentation of the tobacco. At first they are troubled with diarrhœa; later, a peculiar change in the complexion takes place, it assuming a gray color; the blood is very much impoverished, and they are subject to passive congestions. But if we compare these symptoms with those which take place in the workmen engaged in the manufacture of lead and mercury, we find that the latter are by far the more dangerous.

Thackrah, Parent, and others, maintain, on the contrary, that the manufacture of tobacco is not associated with any danger to the workmen engaged therein. They support these statements by publishing statistics which prove that in 4000 workmen, none were very materially affected. The views on the injurious consequences of smoking, and especially of chewing tobacco, are not so different. Most observers admit the possibility of a chronic poisoning by tobacco, following the misuse of strong cigars. The phenomena which are produced hereby, are as follows:

stomatitis; black coloration of the teeth; coryza; chronic bronchitis; emaciation; dyspepsia; chlorosis and anæmia; palpitation of the heart; vertigo; and great irritability of the nerves. Some mention, also, hæmoptysis, apoplexy, amaurosis, senile gangrene, delirium, and mania.

It has been observed that smokers who remain a great deal in ill-ventilated rooms, filled with tobacco smoke, are subject to a considerable enfeeblement of the lower extremities, so that they are obliged either to sit or lie down. At the same time, the sensibility of the skin is materially diminished, and the patient complains of headache and stupor. Fresh air and stimulating treatment remove these symptoms in a few days.

Of late years, Raooth and Santlus have made the effects of tobacco on the nervous system, a subject of special attention. The former communicates the history of a passionate smoker, 52 years of age, who exhibits the following symptoms: since the last 17 years, he has been subject to attacks of dizziness, preceded by nightmares. Sometimes he sees his whole surrounding on the right side turn to the left, or turn in a circle, which requires him to take hold of something, and to lie down on his back. His intellectual faculties are not impaired. These attacks, which occur several times during the day, last only for a few minutes, and leave a sensation of weakness in the lower extremities, so that he cannot venture to walk without assistance. They have increased, lately, in intensity, but remit, often, for a few weeks. His disease was generally believed to be hemorrhoidal congestions. Finally, Raooth saw the patient in one of these attacks; and, noticing the feeble but regular pulse, the paleness of the face, the coldness of the surface, and the clammy perspiration on the skin, was led to believe that these symptoms were due to smoking, and was strengthened in this view by the fact that the patient had smoked several strong cigars shortly before the attack occurred. It finally turned out that whenever the patient quit smoking, the attacks of dizziness grew less in number, and the walking improved, but that the return to this habit brought forth a renewal of his trouble.

Santlus states two cases, in which a considerable disturbance of the nervous system was produced by excessive smoking. The first case relates to a young man 20 years of age, having the following symptoms: change of complexion; loss of appetite and of sleep; lips pale; the whole organism delicate; movements of the body simulating chorea; heart-beat weak and irregular; pain on pressure in the epigastric region; frequent movements of the bowels; and considerable emaciation. The patient imagined that he was going to die with entire hopelessness of a future salvation, causing such a degree of desperation that he tried to commit suicide, and had to be taken to an insane asylum, out of which he returned, after three to four years, entirely cured.

The second case concerns a farmer, 30 years of age, also a passionate smoker, who exhibited the following symptoms: in connection with sleeplessness and great anxiety, he suffered, at times, with difficulty of breathing, and irregular beating of the heart. In these attacks, he destroyed everything within his reach; this period of excitement being followed by one of relaxation. The attending physician finally forbid the patient to smoke, and discovered that by doing so, these attacks grew less in number and intensity, but that a return of his habit brought with it a return of these attacks.

Dawosky states that in the many years he has been practicing, he never met with a single case showing such intensity of symptoms produced by the misuse of tobacco, as Dr. Santlus has described in his two cases above. J. Smith noticed paralysis of the face, following excessive smoking.

Samuel Wright, of Birmingham, besides mentioning the effect which the misuse of tobacco has on the activity of the heart, shows, by his experiments on human beings and on animals, that the nervous system suffers, principally. The main evils which he noticed, were: dullness of the organs of sense; irresolution; nervousness; loss of courage, energy and action. Accompanying these: gastritis; laryngitis; pharyngitis; bronchitis; hæmoptysis; and heart disease.

Siebert has seen palpitation of the heart, gastrodynia, trembling of the extremities, and nocturnal pollutions, in consequence of excessive smoking.

The following is an abstract from a letter which I received several years ago from a friend, who was attacked with similar symptoms as stated above, on account of the misuse of tobacco. He writes:

"In the year 1860 I was troubled a great deal with dizziness, sleeplessness, pains in the head and back, and loss of appetite. By taking liquor, I could get rid of these symptoms, to a great extent, but not entirely. I went, finally, to a watering-place, hoping to obtain a cure, but was disappointed. I consulted a great many physicians, but not one could state the cause of my disease. The symptoms getting gradually worse, it occurred to me that it might be due to my smoking several strong cigars every day, which habit I quit immediately. Great improvement followed, and I am nearly well; but if I smoke a cigar, I am affected just the same as before."

From the description of these different cases, we may come to the following conclusions:

1. That certain persons are subject to chronic poisoning by tobacco, produced by its excessive use.

2. It has not been possible, as yet, to prove which internal or external conditions favor the development of chronic poisoning by tobacco. In proportion to the large number of workmen engaged in the manufacture of tobacco, in proportion to those who smoke or chew tobacco, the number of those who are affected by this disease is very small, indeed, and naturally raises the question how it is possible that the majority of tobacco-smokers are not affected at all, and that others are affected so seriously. There is no doubt that the smoking of cigars has some influence in this respect; for the tobacco which is used for smoking purposes, loses, by fermentation, about two-thirds of its nicotina, which is not the case with that used for cigars; and, consequently, cigars contain three times the quantity of nicotina. Another point of importance, is the quantity of nicotina contained in the different varieties of tobacco. According to Orfila, Schlosing, and others, the average quantity of nicotina contained in the dried leaves, is as follows: Havana, about two per cent.; Maryland, two per cent.; Pas du Calais, five per cent.; Kentucky, six per cent.; Virginia, about seven per cent., etc. Siebert is certainly right in saying that, since a cigar is more liked than an old-fashioned pipe of tobacco, the affections of the nervous system in smokers have greatly increased in frequency.

3. The symptomatology of chronic poisoning by tobacco varies somewhat, as is seen in the cases I have stated; but there are quite a number of symptoms which are present in all, and which resemble those of acute poisoning by nicotina. I will enumerate these symptoms again, according to the organs in which they take place, and especially those which are most prominent.

*A.* Organs of special sense: conjunctivitis, produced, most likely, by the dust of the leaves, or by the smoke; photophobia; optical illusions; amaurosis; and blepharospasmus.

*B.* Skin: this takes on a yellowish hue; it is often cold, and covered with a clammy perspiration; different skin-diseases, as boils, etc., are of not unfrequent occurrence.

*C.* The digestive organs show a series of disturbances, produced by the direct contact with the nicotina present in the saliva swallowed. We have: stomatitis; glossitis; coating of the tongue and teeth; gastrodynia; neuralgia; mesenterica; loss of appetite; dyspepsia; diarrhæa; and, in severe cases, paralysis of the rectum.

*D.* The respiratory organs suffer, also, on account of the direct contact with the poison, the result being: coryza; pharyngitis; bronchitis; hæmoptysis; asthma, etc.

*E.* The organs of circulation present one uniform symptom, which is also noticed in smokers not affected by any other symptom, this being palpitation of the heart, combined with a feeling of anxiety.

*F.* The sexual organs suffer the least of any. It is only in few cases that we find nocturnal pollutions and paralysis vesicæ.

*G.* The nervous system, on the contrary, suffers the most of all: at first we have a series of different forms of hyperæsthesia and neuralgia; the whole skin is subject to a high degree of anæsthesia.

The muscular system seems to be weakened: especially is this the case in the lower extremities, requiring the patient to sit or lie down. Furthermore, the trembling of the limbs, the sensation of being on a floating vessel, when the patient walks on a smooth surface, and the movements of the body resembling chorea, are points of interest.

A very important symptom is the dizziness, associated with sleeplessness, and the sensation of all surrounding objects turning around in a circle.

Disorders of the mind are very frequent, especially the feeling of anxiety, timidity, fearfulness, dullness, loss of energy and courage, with delirium and melancholy, in the worst cases.

Before closing, I want to mention another very important fact, which may facilitate the diagnosis. I refer to the improvement of all these symptoms, following the free use of liquor.

---

The Chemistry of Tetanus.— M. Danilewsky forwards a provisional communication on this subject to the *Centralblatt* of last month, and states that he has arrived at the following conclusions, some of which are already known, whilst others are original: 1. When the circulation of the blood is maintained, the amount of water in active muscle augments, the heart becoming the most watery of all. 2. The quantity of albuminoid substances diminishes in tetanus, though to an inconsiderable extent. The quantity of albuminoids in the heart is much less than in the muscles of the extremities. 3. The alcoholic extract of tetanised muscle contains more nitrogen than that of muscle at rest. The heart contains a very large portion of nitrogen in the alcoholic extract. M. Danilewsky thinks this affords strong evidence of increased disintegration of complex nitrogenous compounds in tetanus. 4. His most recent researches show that in the decomposition of albuminous compounds by means of KOH, compounds containing sulphur, and soluble in warm alcohol, are produced, and hence on *a priori* grounds their presence might be anticipated in the alcoholic extract of warm muscle; and this is fully borne out by analysis. The amount of such compounds cannot, however, as yet be quantitatively determined. The heart again, in this point of view, occupies the first rank, and this furnishes additional evidence of the disintegration of albuminous compounds during muscular activity. 5. Alcoholic extract of muscle contains a phosphorised body (lecithin?) in quantity too large to admit of its coming from the nerves; and it would appear that this also is increased in muscle during long-continued exertion. M. Danilewsky considers that the warm alcoholic extract contains exclusively the products of the regressive metamorphosis of muscle.—*Lancet for August* 31, 1872.—*Mich. University Med. Journal.*

Effect of Bad Air Illustrated.—A striking example, says the *Med. and Surg. Reporter*, of the effects of vitiated air is detailed by Dr. R. A. Smith, of London, in *Nature.* It sets in a clear light the fact that no great diminution of the percentage of oxygen in the air is necessary to make an atmosphere almost deadly to human beings, and the fact that we may be about as ignorant of the noxious quality of the air we are breathing, even while we are taking it into our lungs, as we are susceptible of injury from it. In some of his experiments he used an air-tight chamber, the atmosphere of which he vitiated by burning candles in it. On one occasion a young lady, who was anxious to make the experiment, got into this enclosure just as the candles were put out, and at the end of five minutes, during which she made light of the difficulty, it was necessary to help her out, the partial asphyxiation having taken place in an atmosphere in which the percentage of oxygen was about 19.0.—*Lancet for August* 31, 1872. *Mich. University Med. Journal.*

Case of Beriberi.—An interesting case of beriberi, the symptoms of which at one time strongly resembled those of aortic aneurism, is detailed in the *Madras Monthly Journal of Medical Science*, by G. Williamson, M. D., Assistant-Surgeon of the 19th M. N. I. At last advices, the patient, private, aged 33 years, was on sick leave to his native village, and doing well. The formula most beneficial to him was the following: R. Potas. acet., gr. x.; tinct. scillæ, ♏x.; spir. nitric æther, ♏xx.; liq. ammon. acet., ℥ij.; Camph. mixt., ℥i. To be taken every three hours.

